# Burial Reform Association

**Published:** 1888-01

**Authors:** 


### BURIAL REFORM ASSOCIATION.

The recent organization of a Burial Reform Association in New York, of which Bishop Potter, was chosen President, was a move in the right direction.

The objects of the association are stated to be:

"1. To encourage burial in perishable coffins in the simple earth.
"2. To simplify and cheapen funerals and mourning ceremonies.
"3. To secure large and ample tracts of suitable grounds for burial purposes."

The following specific reforms were adopted as the sentiments of the association:

1. The exercise of economy and simplicity in everything pertaining to the funeral.
2. The use of plain hearses.
3. The disuse of crape, scarfs, feathers, velvet trappings, and the like.
4. The avoiding of all unchristian and heathen emblems, and of the use of any floral decorations beyond a few cut flowers.
5. The discouraging of all eating and drinking in connection with funerals.
6. The discouragement of any but immediate members of the family accompanying the body to the grave.
7. The dispelling of the idea that all the club or society money must be spent on the funeral.
8. The early interment of the body in soil sufficient and suitable for its re-solution into its ultimate elements.
9. The use of such materials for the coffin as will rapidly decay after burial.
10. The substitution of burial plots for family vaults.
11. The encouragement on sanitary grounds of the removal in crowded districts of the body to a mortuary, instead of retaining it in the rooms occupied by the living.
12. The impressing upon officers of public charities and correction of the claims of the poorest to proper and reverent burial.

After some discussion the use of crape as a symbol of mourning was voted down, so that it is to be hoped that this costly and senseless inheritence of barberism, may yet be dispensed with.

The society is to be entirely undenominational in its work, and any Christian can join on payment of one dollar.

